# Flexible TEMPO-cellulose/silver selenide nanocomposites with advanced optical, electrical, and antimicrobial performance

**DOI:** 10.1039/d5ra09670g

**Published:** 2026-02-25

**Authors:** Ahlam I. Al-Sulami, Fatimah Mohammad H. Al Sulami, Reema H. Aldahiri, Abdelilah Lahmar, Jacem Zidani, Talaat A. Hameed

**Affiliations:** a College of Science, Department of Chemistry, University of Jeddah Jeddah 21589 Saudi Arabia; b Laboratoire de Physique de la Matière Condensée, Université de Picardie Jules Verne Amiens UR 2081 France; c Solid-State Physics Department, Physics Research Institute, National Research Centre 33 El Bohouth St., Dokki Giza 12622 Egypt Talaathameed83@gmail.com Talaathamid@yahoo.com

## Abstract

Transforming dielectric polymers into conductive or semiconductive materials opens new avenues for advanced and unprecedented applications. Herein, flexible films were fabricated from TEMPO-oxidized cellulose nanofibers/silver selenide (T-CNF/Ag_2_Se) nanocomposites. Ag_2_Se particles were *in situ* prepared in the presence of TEMPO-oxidized cellulose nanofibers to limit the Ag_2_Se formation within the nanopores of the TEMPO-oxidized cellulose nanofibers. XRD and FTIR patterns verified the effective embedding of Ag_2_Se nanoparticles within the T-CNF matrix, where Ag_2_Se crystallized exclusively in the orthorhombic β-Ag_2_Se phase. For optoelectronic applications, the optical features were investigated, and Ag_2_Se has a great impact on transmittance, reflectance, optical band gap, and Urbach energy of CNF. The transmittance was reduced from 10% to 2% in the visible region, while the optical band gap dropped from 4.46 eV for CNF to 2.65 eV for CNF/Ag_2_Se I. Compared with pure CNF, the CNF/Ag_2_Se I nanocomposites showed broader M″ peaks that shifted towards higher frequencies, indicating enhanced charge-carrier dynamics due to the additional conductive pathways introduced by the Ag_2_Se nanoparticles. At 313 K, conductivity followed the order of CNF < CNF/Ag_2_Se III < CNF/Ag2_S_e < CNF/Ag_2_Se II < CNF/Ag_2_Se I, with the conductivity increased by three orders of magnitude for CNF/Ag_2_Se I compared with that for the pure CNF. The antimicrobial performance of CNF/Ag_2_Se at different concentrations was evaluated, and it exhibited high toxicity against *E. coli*, *S. typhimurium*, and *C. albicans*, while *S. mutans* exhibited more resistance against the nanocomposite materials.

## Abstract 2

Ag_2_Se was *in situ* prepared in TEMPO-oxidized cellulose nanofibers for optical , electrical and antimicrobial applications.

## Graphical abstract

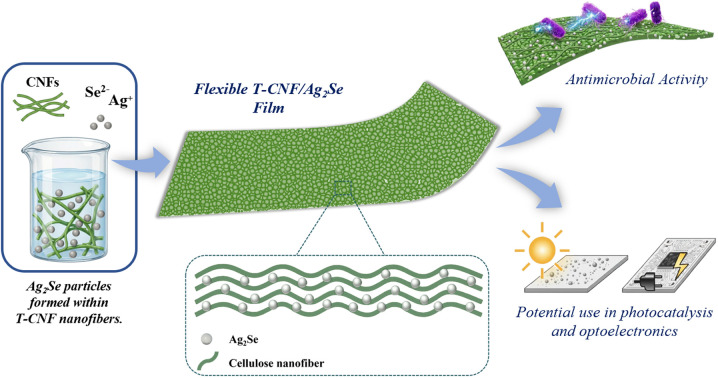

## 1 Introduction

Transition metal chalcogenides (TMCs) have attracted increasing interest in recent periods.^1,2^ Various TMCs, including cobalt selenide, molybdenum selenide, tungsten selenide, copper selenide, and silver selenide, have been applied in numerous fields, such as batteries, supercapacitors, and sensors. Since TMCs are highly electrically conductive, electroactive, and stable, they are very useful in electrical applications.^3^ TMCs containing selenium exhibit exceptional electrocatalytic performance due to the ability of selenium to improve a metal's electronic composition and prevent the electrochemical oxidation of elements in most transition metal chalcogenides. Semiconductors, such as Ag_2_Se, are utilized currently for their excellent optical, electrical, and chemical properties, especially for their narrow and direct band gap (0.07–0.15 eV for Ag_2_Se), reasonable chemical stability, thermal stability, and exceptional emission in the second near-infrared region.^4,5^ As a result, semiconductors are utilized in biomedical applications, such as *in vivo* imaging, solar cells, thin-film transistors, optical detectors, and optical filters.^6,7^ Several studies have shown that the Ag_2_Se nanoparticles inhibit the activity of Gram-positive and Gram-negative resistant bacteria, along with causing low cytotoxicity in cells.^8,9^ Gregory Von White *et al.* examined the effect of micromolar concentrations (25 µg mL^−1^) of silver nanoparticles on muscle cells and fibroblasts. No significant decrease in cellular proliferation was observed in vascular smooth muscle cells and 3T3 fibroblasts, suggesting that silver nanoparticles prepared with garlic extract are possible candidates for implementation in the biomedical applications.^9^ Furthermore, silver/chitosan nanocomposites have been reported as promising candidates for cell proliferation and cell adhesion.^10^

Silver selenide (Ag_2_Se) has unique and important properties, and consequently, it is usually employed in gas sensors, memory devices, electrochemical sensors, and batteries.^11,12^ Ag_2_Se normally exists in two separate forms: β-Ag_2_Se at low temperatures and α-Ag_2_Se at high temperatures. Ag_2_Se is a small-band-gap material that can be utilized as a photosensitive agent in photography films. Additionally, silver selenide acts as a type of semiconductor and hence can be useful in electrochemistry.^13^ It exhibits a small band gap, and its nonstoichiometric derivatives exhibit significant positive and negative magnetoresistance properties. The adaptable, distinctive architecture and characteristics of Ag_2_Se nanomaterials make them suitable for device functionality improvement.^11^

Nanocellulose materials, such as cellulose nanofibers (CNFs), and their derivatives have been prepared from the most abundant and renewable biopolymer on earth, *i.e.*, cellulose. These biomaterials have distinctive physicochemical properties, such as high tensile strength, large elastic modules, and low density. CNFs with a high surface-to-volume ratio have been prepared by the (2,2,6,6-tetramethylpiperidiniyl-1-oxyl)-oxidation (TEMPO) method, which causes a lesser degree of degradation to the amorphous structure than the acidic hydrolysis process of CNCs. They are accordingly applied in various applications, such as reinforcing fillers, optical materials, electroconductive materials, and biomedical materials.^14^ For example, a flexible thermoelectric paper was prepared from bacterial cellulose/silver selenide nanocomposites. The results showed that the *in situ* synthesis produces submicrosized Ag_2_Se particles with a narrow size distribution and homogeneous dispersion in the nanofiber network.^15^ Several trials have been carried out for the investigation of cellulose/silver selenide composites. For example, a thermoelectric paper was prepared from bacterial cellulose/silver selenide (BC/Ag_2_Se) nanocomposites through an *in situ* technique. The Ag_2_Se particles with a narrow size distribution were homogeneously distributed in the BC network. The results showed a significant enhancement of the TE properties, with an electrical conductivity of 23 000 S m^−1^ and a Seebeck coefficient of −167 µV K^−1^ at 400 K. The power factor recorded was 642 µW mK^−2^ at 400 K, a high value compared to those in other flexible TE research.^15^

The *in situ* incorporation of silver selenide with TEMPO-oxidized cellulose nanofibers (T-CNFs) represents a promising strategy for developing flexible, sustainable, and multifunctional nanocomposite films that address the intrinsic limitations of pristine Ag_2_Se. While Ag_2_Se exhibits excellent electrical, optical, and electrocatalytic properties, its practical application is often restricted by its brittle nature, limited processability, and poor mechanical stability.^16^ Embedding Ag_2_Se within a T-CNF matrix enables the uniform dispersion of the semiconductor nanophases within a mechanically robust, lightweight, and renewable scaffold, resulting in free-standing and flexible films with enhanced structural integrity. Moreover, the abundant surface functional groups and high surface-to-volume ratio of T-CNFs promote strong interfacial interactions and facilitate efficient charge transport while suppressing electron–hole recombination at the Ag_2_Se interfaces.^17^ This synergistic architecture allows tunable optical absorption in the visible region at relatively low Ag_2_Se loadings, improving photocatalytic efficiency while minimizing material consumption and potential cytotoxicity.^18^ In addition, the porous and interconnected T-CNF network enhances ion diffusion and electron mobility, rendering the nanocomposite films attractive for energy-related applications, such as passivation layers and electrode components in supercapacitors and other storage devices.^19^ The incorporation of Ag_2_Se further imparts antimicrobial functionality, expanding the applicability of the films to environmental and biomedical domains.^20^ Therefore, T-CNF/Ag_2_Se nanocomposite films constitute a versatile material platform that combines mechanical flexibility, optimized charge dynamics, optical tunability, and antimicrobial activity within a single sustainable system.

In this study, CNF/Ag_2_Se composite films were successfully synthesized and systematically characterized. Incorporating silver selenide (Ag_2_Se) into the cellulose nanofiber (CNF) matrix leads to the formation of a flexible polymeric film, offering significantly broader applicability compared to pure Ag_2_Se, including potential use as a passivating layer in supercapacitors and other energy-storage devices. The CNF matrix not only provides mechanical flexibility but also suppresses electron–hole recombination in Ag_2_Se by acting as a barrier, thereby improving the charge-separation efficiency. Remarkably, only small amounts of Ag_2_Se are required to achieve a visible-range optical band gap, which enhances the photocatalytic performance of the films under light irradiation. Beyond optical and electrical properties, the CNF/Ag_2_Se films demonstrate promising antimicrobial activity, making them suitable for multifunctional applications, including environmental remediation and biomedical coatings. For the first time, this work presents a comprehensive investigation of the structural, compositional, optical, electrical, and antimicrobial characteristics of the CNF/*x*Ag_2_Se films, highlighting their potential as versatile, high-performance materials for next-generation devices.

## 2 Experimental part

### 2.1 Materials

Ascorbic acid (99.0%) and silver nitrate (AgNO_3_) (99.8%) were purchased from Sigma-Aldrich. All the chemicals were used as received without further purification. Yeast extract powder was purchased from Himedia.

### 2.2 TEMPO oxidation of cellulose nanofiber

**Fig. 1:**
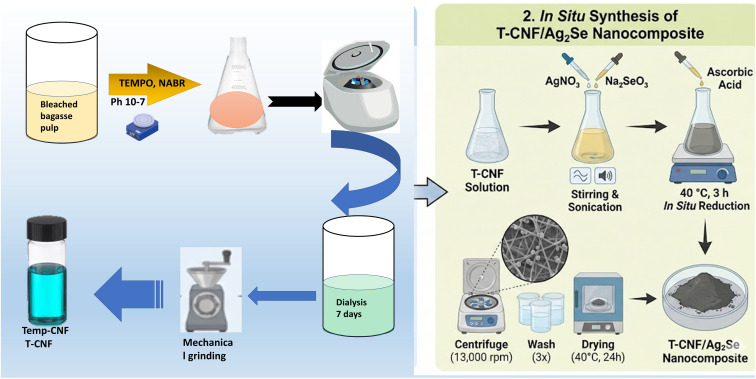
Schematic of the synthesis and application of T-CNFG and the CNF/Ag_2_S polymer nanocomposites.

As previously described, cellulose nanofibers were synthesized from bleached bagasse pulp by TEMPO-oxidation and mechanical defibrillation using a grinder (Masuko Sangyo Co. Ltd, Japan). A solution containing 0.16 g of TEMPO and 1.6 g of sodium bromide in 600 mL of distilled water was added to 4 g of bleached bagasse pulp. After adding 60 mL of sodium hypochlorite, a 0.02 M NaOH solution was used to adjust the pH to 10; it then dropped to 7 after the oxidation process. The prepared TEMPO-oxidized cellulose was separated by centrifuging the solution at 11 000 rpm. After careful washing with water, the suspension was dialyzed for 7 days against deionized water. Mechanical defibrillation was subsequently used to obtain TEMPO-oxidized cellulose nanofiber (TEMPO-CNF). TEMPO-CNF has a carboxylate content of 1.3 ± 0.3 mmol g^−1^, as determined by electric conductivity titration. The preparation procedure is schematically illustrated on the left side of Fig. 1.

### 2.3 Cellulose nanofibers/Ag_2_Se preparation

CNF/Ag_2_Se was fabricated by the *in situ* synthesis of the Ag_2_Se nanoparticles within the structure of CNF. To prepare T-CNF/Ag_2_Se nanocomposites, ascorbic acid was used as a reducing agent, and T-CNF was used as a supporting biopolymer. For this purpose, 10 mL of T-CNF was poured into 100 mL flasks containing 40 mL of distilled water. Then, 1 mL of silver nitrate (AgNO_3_) and 0.5 mL of sodium selenite (Na_2_SeO_3_) containing the amounts listed in Table 1 were added to the solution while shaking on a magnetic stirrer, followed by sonication. The ascorbic acid solution was added under continuous stirring at 40 °C for 3 hours. The T-CNF/Ag_2_Se nanocomposites were centrifuged at 13 000 rpm and washed three times using distilled water. Finally, the precipitate was dried at 40 °C in an oven for 24 h, as illustrated in righ side of Fig. 1

**Table 1:** Code of samples with the amount of CNF, AgNO_3_, and Na_2_SeO_4_ (g)

NC	CNF (mL)	AgNO_3_ (g)	Na_2_SeO_3_ (g)	Ag_2_Se/CNF	CNF	Ag_2_Se
CNF	10	—	—	CNF	100	00.00
CNF/Ag_2_Se I	10	0.0100	0.00500	1 : 1	100	100.00
CNF/Ag_2_Se II	10	0.0050	0.00250	1 : 2	100	50.00
CNF/Ag_2_Se III	10	0.0025	0.00125	1 : 3	100	33.33

### 2.4 Antimicrobial evaluation by agar well diffusion method

The antimicrobial assessments of the CNF, CNF/Ag_2_Se I, CNF/Ag_2_Se II, and CNF/Ag_2_Se III nanocomposite complexes were qualitatively investigated using the agar well diffusion method, with minor modifications, against four pathogenic microbes: Gram-negative bacteria *Escherichia coli* ATCC 25922 (*E. coli*) and *Salmonella typhimurium* ATCC 14028 (*S. typhimurium*), Gram-positive bacteria *Streptococcus mutant* ATCC 25175 (*S. mutans*), and yeast *Candida albicans* ATCC 10231 (*C. albicans*).^21^ All of the pathogenic microbes were purchased from the American Type Culture Collection (ATCC). The pathogenic microorganisms were initially cultivated in a test tube containing 5 mL of Mueller–Hinton broth composed of (%w/v): 0.2 beef extract, 1.75 acid hydrolysate of casein, and 0.15 starch. The cultures were incubated at 37 °C for 24 h under shaking conditions at 200 rpm. After incubation, approximately 100 µL of each microbial suspension (10^8^ CFU mL^−1^) with approximately equal concentration or density with 0.5 McFarland standards was spread on a solidified Mueller Hinton agar medium (agar 2%). Four perforations (3 mm in diameter) were made using a sterile glassy borer, and each well was filled with 70 µL of the tested nanocomposite. The plates were incubated at 4 °C for 1 hour to allow the tested nanocomposite to diffuse and temporarily inhibit the pathogenic microbes, followed by incubation at 37 °C for one day. Finally, the antimicrobial activity was determined by measuring the developed inhibition zone diameter (including the well) after the incubation period. All experiments were conducted in triplicate, and the mean values were reported.

### 2.5 Instrumentation

The crystalline structure of both the pristine cellulose nanofibers and silver selenide-incorporated cellulose nanofibers was investigated using a Philips X'pert diffractometer employing copper K_α_ radiation with a wavelength of 1.540 Å. Nanoscale morphological examination of the unmodified and Ag_2_Se-functionalized cellulose nanofibers was performed using a JEOL1230 transmission electron microscope operating at an accelerating voltage of 200 kV. Surface morphology and structural features were further analyzed through field-emission scanning electron microscopy utilizing a Quanta FEG 250 instrument manufactured by FEI. The optical characteristics, including light transmission and reflection behavior, were evaluated across a broad spectral range spanning 190 to 2500 nanometers at ambient temperature. These measurements were obtained using a JASCO V-570 dual-beam spectrophotometer. The dielectric properties were characterized employing an Alpha-A Broadband Dielectric Spectroscopy system (concept 40 model from Novocontrol Technologies). Measurements were conducted across a frequency spectrum of 0.1 Hz to 10 MHz while varying the temperature between 273 and 313 K. For these measurements, film specimens were positioned between a pair of gold-coated electrodes, each having a diameter of 20 millimeters.

## 3 Results and discussion

### 3.1 Structural investigation of the T-CNF/Ag_2_Se films

#### 3.1.1 Crystallographic study

**Fig. 2:**
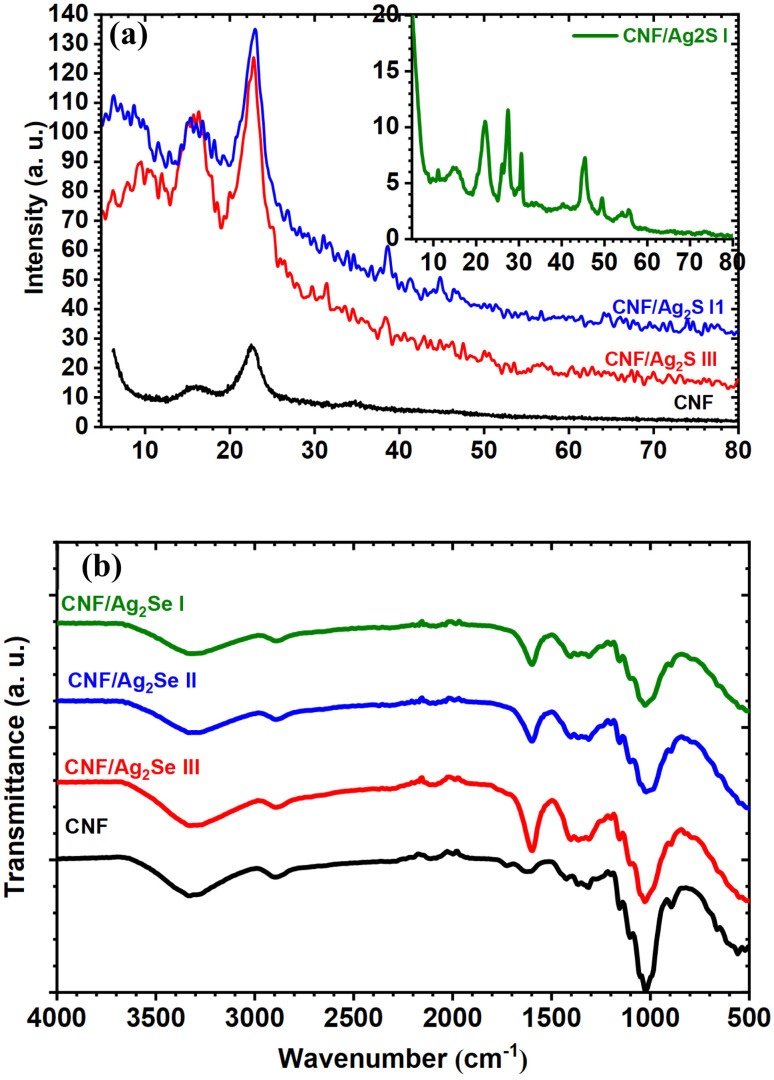
(a) X-ray diffraction spectra and (b) FT-IR spectra of CNF, CNF/Ag_2_Se III, CNF/Ag_2_Se II, and CNF/Ag_2_Se I.

The phase structures of the pristine T-CNF and Ag_2_Se-filled CNF with different concentrations were studied by X-ray diffraction analysis, as depicted in Fig. 2a, and the inset of the figure shows the results for CNF/Ag_2_Se I, which has a high concentration of Ag_2_S. The XRD patterns of the pristine T-CNF revealed the characteristic cellulose crystallinity, showing diffraction peaks at 16.4° and 22.5°, corresponding to the (101) and (002) planes. After incorporating Ag_2_Se at different weight percentages, additional sharp reflections appeared at 30.9°, 39.9°, 43.72°, 48.05°, and 78.08°, which are indexed to the (102), (031), (032), (004), and (116) planes of β-Ag_2_Se, as referenced in JCPDS card No. 24-1041. The absence of any extra or secondary diffraction peaks confirms that the Ag_2_Se nanoparticles formed within the T-CNF matrix possess high phase purity. The CNF/Ag_2_Se I film shows a sharp peak, owing to a high concentration of Ag_2_Se. Therefore, the XRD evidences the strong intercalation between CNF and silver selenide.

These observations indicate that the Ag_2_Se crystallized exclusively in the orthorhombic β-Ag_2_Se phase. Based on these reflections, the corresponding lattice parameters (*a*, *b*, and *c*) were determined using the standard calculation formula provided in the ref. 18.1
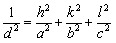


The obtained values of the lattice parameters are *a* = 4.313 Å, *b* = 7.042 Å, and *c* = 7.754 Å, which are very consistent with the reported JCPDS No. (*a*, *b*, and *c*) and some previously reported values.^22^

Changes in the intensity of the diffraction peaks indicate variations in both grain size and the degree of crystallinity (*X*_c_). Thus, the degree of crystallinity can be calculated according to the following relations:^23^

2 *X*_c_ = *S*/*S*_o_ × 100,

where *S* and *S*_o_ imply the area under the crystalline peaks and the total area under the diffraction pattern, respectively. It was found that the degrees of crystallinity were 15.23%, 22.36%, 28.56%, and 48.23% for CNF, CNF/Ag_2_Se I, CNF/Ag_2_Se II, and CNF/Ag_2_Se III, respectively. The *in situ* formation of Ag_2_Se within the CNF polymer matrix fills regions that were originally amorphous, leading to a reduction in free volume and amorphous disorder while increasing the overall crystalline content of the material.

It is clear that the *in situ* incorporation of Ag_2_Se significantly alters both the peak intensities and the full width at half maximum (FWHM), indicating changes in grain size. Therefore, the Debye–Scherrer equation was employed to determine the corresponding grain size.^24,25^3
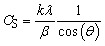
Here, *t* expresses the thickness of the films (1 µm). The grain sizes were found to be 15.24, 22.23, and 30.45 nm for CNF/Ag_2_Se III, CNF/Ag_2_Se V, and CNF/Ag_2_Se II, respectively. The observed increase in the grain size with increased Ag_2_Se content can be attributed to the enhanced availability of the Ag_2_Se precursor species, which promote more effective nucleation, followed by accelerated crystal growth. As the Ag_2_Se loading increases, the nanoparticles progressively occupy amorphous regions within the CNF matrix, reducing polymeric disorder and providing a more favorable environment for crystal development. The higher concentrations of the Ag^+^ and Se^2−^ ions also improve their mobility and collision frequency during formation, facilitating the growth of larger crystallites. In addition, at elevated loadings, neighboring Ag_2_Se nanoparticles tend to coalesce, further contributing to the increase in the grain size. Collectively, these factors support the formation of larger, more ordered Ag_2_Se crystallites with increasing filler content.

#### 3.1.2 FTIR spectroscopy

Fig. 2b demonstrates the FTIR spectrum of T-CNF. The spectrum reveals the characteristic peaks of cellulose chains. The broad peak at 3332 cm^−1^ is assigned to the –OH stretching groups, while the peak at 2919 cm^−1^ reveals the stretching vibration of the C–H and CH_2_ groups. The peak at 1015 cm^−1^ represents the stretching vibration of CH_2_–O–CH_2_. The weak band at 1739 cm^−1^ is assigned to the C=O stretching vibrations, which originated from the TEMPO oxidation process. Slight shifts and intensity changes in the O–H and C–O–C regions indicate interactions between Ag_2_Se nanoparticles and the CNF matrix, suggesting successful incorporation. Increasing the Ag_2_Se content (from I to III) enhances these changes, reflecting stronger interactions and the modification of the polymer structure.

### 3.2 HRTEM study

**Fig. 3:**
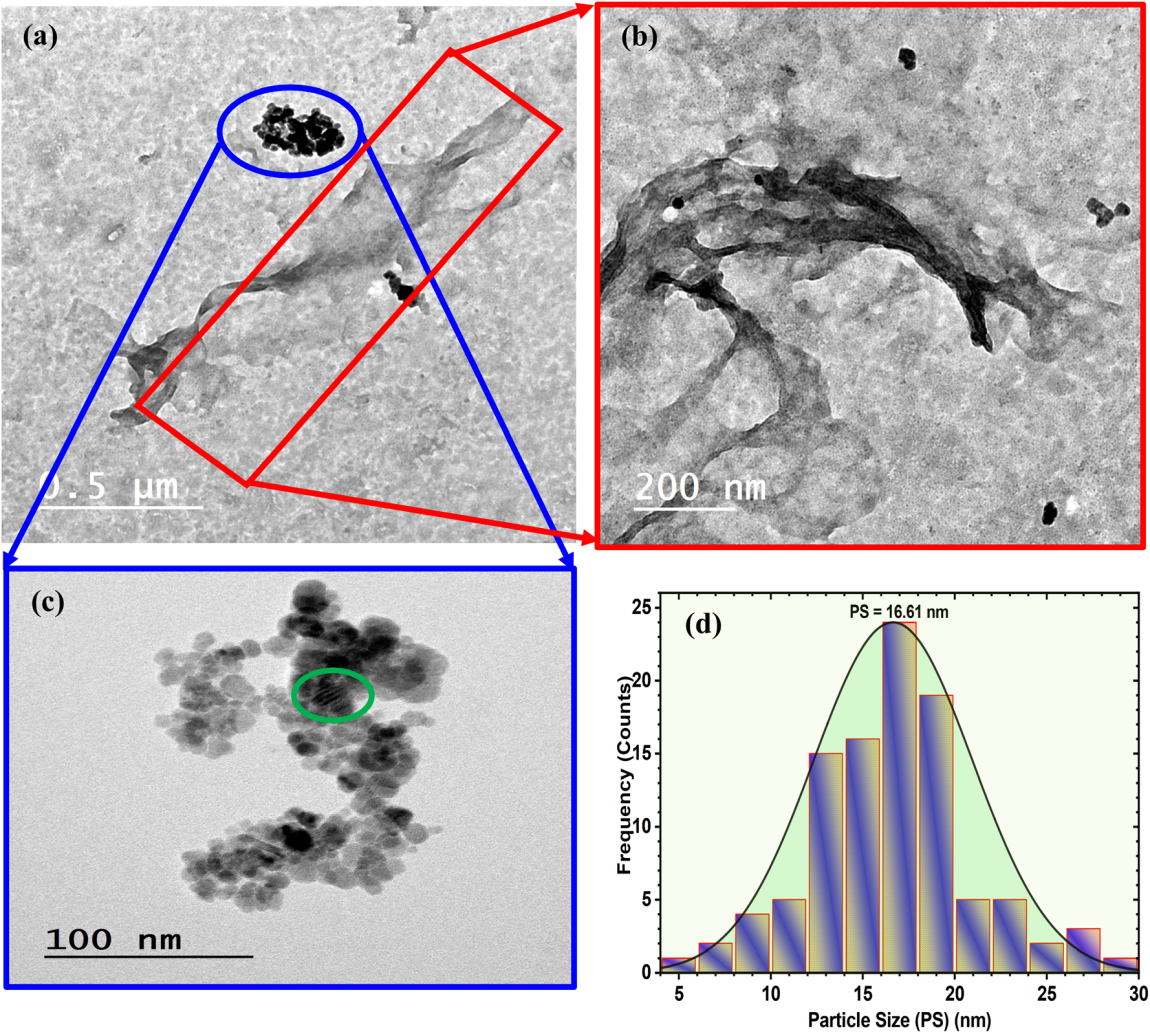
(a) Low- (b and c) high-magnification HRTEM images, and (d) particle-size distribution histogram fitted by lognormal distribution function, including all the goodness-of-fit parameters for CNF : Ag_2_Se III.

The morphology of the Ag_2_Se nanoparticles in the CNF film was investigated using TEM, as shown in Fig. 3. The low magnified image (Fig. 3a) displays the presence of polymeric samples nanofibers (T-CNF) (square rectangle) alongside some agglomeration of inorganic clusters (Ag_2_Se) (blue oval). The CNF displays a distinctive fibrillar structure with an elongated, ribbon-like morphology (magnified image, Fig. 3b). The fibers appear to have a width in the nanometer range, which is typical for mechanically or chemically isolated cellulose nanofibers. In addition, the image reveals a somewhat twisted or curved configuration of the nanofiber, which is characteristic of CNF due to the hierarchical structure of cellulose and the isolation process. The blue oval clearly shows the presence of the Ag_2_Se nanoparticles, which adopt spherical to quasi-spherical shapes (magnified image, Fig. 3c). The Ag_2_Se nanoparticles exhibit a relatively uniform size distribution, appearing to be in the range of approximately 5–20 nm in diameter, agreeing with the XRD calculations. The nanoparticles form clustered or aggregated regions along with what appears to be the CNF structure. This clustering pattern suggests that the Ag_2_Se particles preferentially nucleate and grow on or within the cellulose nanofiber network, likely due to the abundant hydroxyl groups on the cellulose surfaces that can serve as nucleation sites. The distribution pattern suggests good interaction between the Ag_2_Se nanoparticles and the CNF matrix. The particles appear to be embedded within or attached to the nanofiber structure rather than existing as separate phases, indicating successful incorporation. The filler particle size was evaluated from the histogram presented in Fig. 3d. The histogram shows a relatively narrow, near-Gaussian (normal) distribution, which is indicated by the bell-shaped curve overlaid on the bar chart. This suggests a well-controlled synthesis process with consistent nucleation and growth conditions. The particle sizes span approximately from 5 to 30 nm, with the majority of particle sizes falling between the range of 12 and 22 nm. This relatively tight distribution is favorable for applications requiring uniform properties across the nanocomposite.

### 3.3 Morphology investigation

**Fig. 4:**
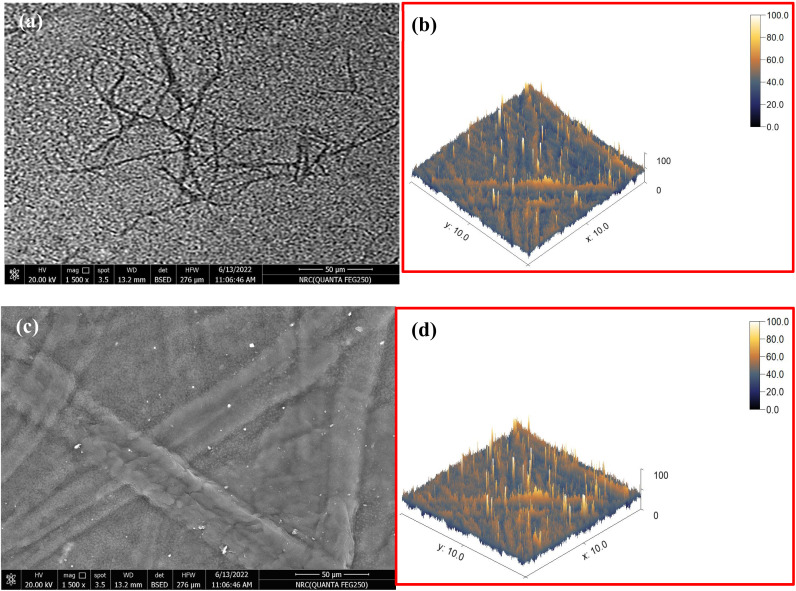
(a and c) SEM images of CNF and CNF/Ag_2_Se III, respectively, and (b and d) corresponding 3D SEM micrographs.

Three different films of cellulose nanofibers/Ag_2_Se with different Ag_2_Se contents were coded as CNF/Ag_2_Se I, CNF/Ag_2_Se II, and CNF/Ag_2_Se III. The microstructure of the CNF/Ag_2_Se films is shown in Fig. 4. The SEM micrograph of the pristine CNF sample (Fig. 4a) shows a highly entangled network of long and ultrafine cellulose nanofibers with smooth surfaces and a uniform morphology. The fibers form a random web-like structure typical of TEMPO-oxidized cellulose, with no observable particulate deposits. In contrast, the Ag_2_Se-doped CNF sample (Fig. 4c) exhibits a noticeably modified surface morphology. The nanofibers appear thicker and more textured, indicating the successful nucleation and growth of the Ag_2_Se nanoparticles on the CNF backbone. Numerous bright nanoscale spots are distributed along the fibers, confirming the presence of Ag_2_Se particles. The overall structure becomes denser and more consolidated compared to the pure CNF network, reflecting the strong interaction between Ag_2_Se and the cellulose matrix. This unique uniform size and distribution of the CNF/Ag_2_Se nanocomposite benefits from the *in situ* synthesis of Ag_2_Se in the presence of CNF, which limits the growth or clustering of the Ag_2_Se nanoparticles.

### 3.4 Optical properties of T-CNF/Ag_2_Se films for optoelectronic applications

#### 3.4.1 Transmittance and reflectance

**Fig. 5:**
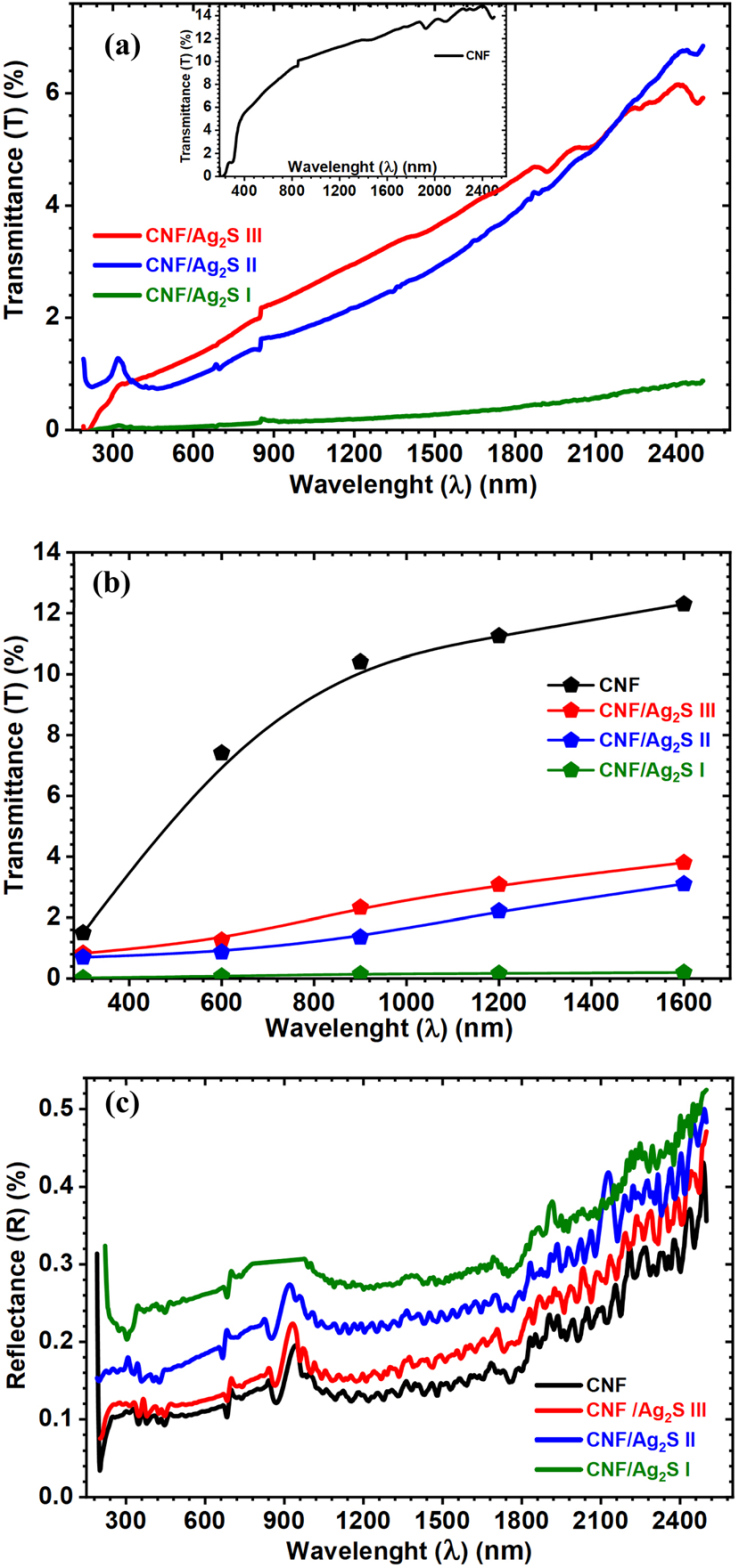
(a) Spectral variation in transmittance, and the inset figure shows the results for the pure CNF. (b) Variation in transmittance at a specific wavelength and (c) spectral variation of reflectance for CNF, CNF/Ag_2_Se III, CNF/Ag_2_Se II, and CNF/Ag_2_Se I.

In general, the examination of optical properties is very effective in exploring the variation of electronic and band structure after the filling process. In addition, the optical properties are related to the electrical properties, where the optical properties determine the optical band gap, which is a function of the electrical properties.^26^ To that end, the synthesized CNF, CNF, CNF/Ag_2_Se III, CNF/Ag_2_Se II, and CNF/Ag_2_Se I were optically characterized through UV-vis-NIR spectroscopy. Fig. 5a shows the spectral variation in the transmittance of the pure CNF and its different Ag_2_Se-filled CNF materials with different concentrations. Pure CNF has the highest transmittance over a wide range of wavelengths, whereas the CNFB shows the highest absorbance. The paper films show a very low transmittance compared to the state-of-the-art TEMPO-oxidized CNF films, which often exhibit a very high optical transmittance (>85%). The relatively low transmittance (∼14%) observed for the pure CNF film in the present study arises from fundamental differences in the raw material source, processing route, and film microstructure, rather than experimental error. First, it should be emphasized that the CNF used in this work was derived from bagasse pulp *via* mechanical/chemical treatment without TEMPO-mediated oxidation. Compared to TEMPO-CNF, bagasse-derived CNF commonly retains (i) partial microfibril bundles due to incomplete defibrillation; (ii) residual hemicellulose and trace lignin content, even after bleaching; and (iii) a broader fiber diameter distribution, extending into the tens of nanometers. These factors significantly enhance light scattering, which is the dominant cause of opacity in cellulose-based films. In particular, residual lignin and hemicellulose act as UV-visible chromophores, while larger fibrillar aggregates produce Mie scattering, both of which strongly reduce optical transmittance. Otenda and co-workers^27^ demonstrated that the bagasse-derived CNF/PVA showed a very low transmittance of about 8% and increased as the thickness decreased. Second, the film fabrication method also plays a critical role. The CNF films were prepared by solution casting and drying, which typically leads to dense hydrogen-bonded networks, surface roughness and thickness inhomogeneity, and microvoids and inter-fiber interfaces. Such structural features further increase light scattering and reduce transparency, even when the chemical purity is relatively high. Zeng *et al.* reported that CNF prepared *via* a combined process of enzymatic hydrolysis and grinding exhibited very low transmittance, with a maximum transmittance of approximately 20%, which is consistent with our results.^28^ Third, the film thickness used in this study is significantly larger than that commonly reported for highly transparent TEMPO-CNF films. Since optical transmittance decreases exponentially with thickness, this alone can account for the substantial reduction in the measured transmission. This behavior has been confirmed by previous works.^27^ CNF/Ag_2_Se I shows the lowest transmittance value. For pure CNF, the transmittance is relatively high (about 14%), and it increases gradually until the end of the spectrum, revealing the low absorbance of pure CNF in the visible and NIR regions. The transmittance of the Ag_2_Se-modified CNF shows a clear linear dependence on the wavelength over the entire wavelength range; however, its transmittance is lower than that of the pure CNF. Fig. 5b shows the value of transmittance at a specific wavelength to show how the transmittance drops in the order of CNF > CNF/Ag_2_Se III > CNF/Ag_2_Se II > CNF/Ag_2_Se I. These results reflect the fact that the inclusion of Ag_2_Se reduces the transmittance because it generates localized states that elevate the absorbance and lower the transmittance. Further, the reduced transmittance can be attributed to an increase in the absorption cross-section and number of light-scattering centers introduced by the Ag_2_Se nanoparticles as their concentration increases. Additional contributions from the increased effective film thickness and nanoparticle aggregation likely amplify the reduction in transmittance for high-loading samples. All the films display a strongly sharp absorption edge around 250 to 450 nm, which embodies the main transition of the HOME and corresponds to the π → π* transition resulting from the benzenoid unit and the quinonoid excitation band, respectively. The onset of the absorption edge shifts to a higher wavelength depending on the content of Ag_2_Se. Fig. 5c portrays the variation of the reflectance of the pristine CNF and Ag_2_Se-modified CNF. The reflectance of the CNF/Ag_2_Se films increases systematically with the Ag_2_Se loading, following the order of CNF < CNF/Ag_2_Se III < CNF/Ag_2_Se II < CNF/Ag_2_Se I. This behavior is attributed to the high refractive index of Ag_2_Se, which enhances the Fresnel reflection at the film–air interface, together with the increased number of scattering centers introduced by the nanoparticles. Higher Ag_2_Se concentrations generate stronger diffuse backscattering and rougher film surfaces, both of which contribute to the increased reflectance across the UV-NIR region. The significant rise in reflectance at long wavelengths (>1500 nm) is consistent with enhanced scattering and NIR absorption associated with the Ag_2_Se nanoparticles.

#### 3.4.2 Optical band gap and Urbach energy

The optical band gap represents the energy difference between the HOMO band and LUMO, upon which many applications are decided, like optoelectronics and photocatalysis. The Urbach energy (*E*_u_) is a good representation of disorders as a function of localized states created in the forbidden region (SCR), and the relation between the optical band gap and Urbach energy is always inverse. The optical band gap can be assessed on the basis of the Tauc's equation, as mentioned below:^29–31^

4 *αhν* = *A*(*hν* − *E*_g_)^*p*^

where *ν* refers to the frequency of, *h* signifies Planck's constant, *E*_g_ denotes the optical band gap, and the *A* constant relies on the effective masses associated with the band. *p* refers to the complex parameter that describes the type of transition. The values of *p* were evaluated using the method described in our previous study,^32^ and the obtained results were approximately ½, confirming that the samples exhibit a direct allowed electronic transition. *A* in the above expression is the absorption coefficient that can be calculated from the absorbance (*A*) and thickness (*d*) by the following formula.5
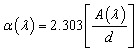


**Fig. 6:**
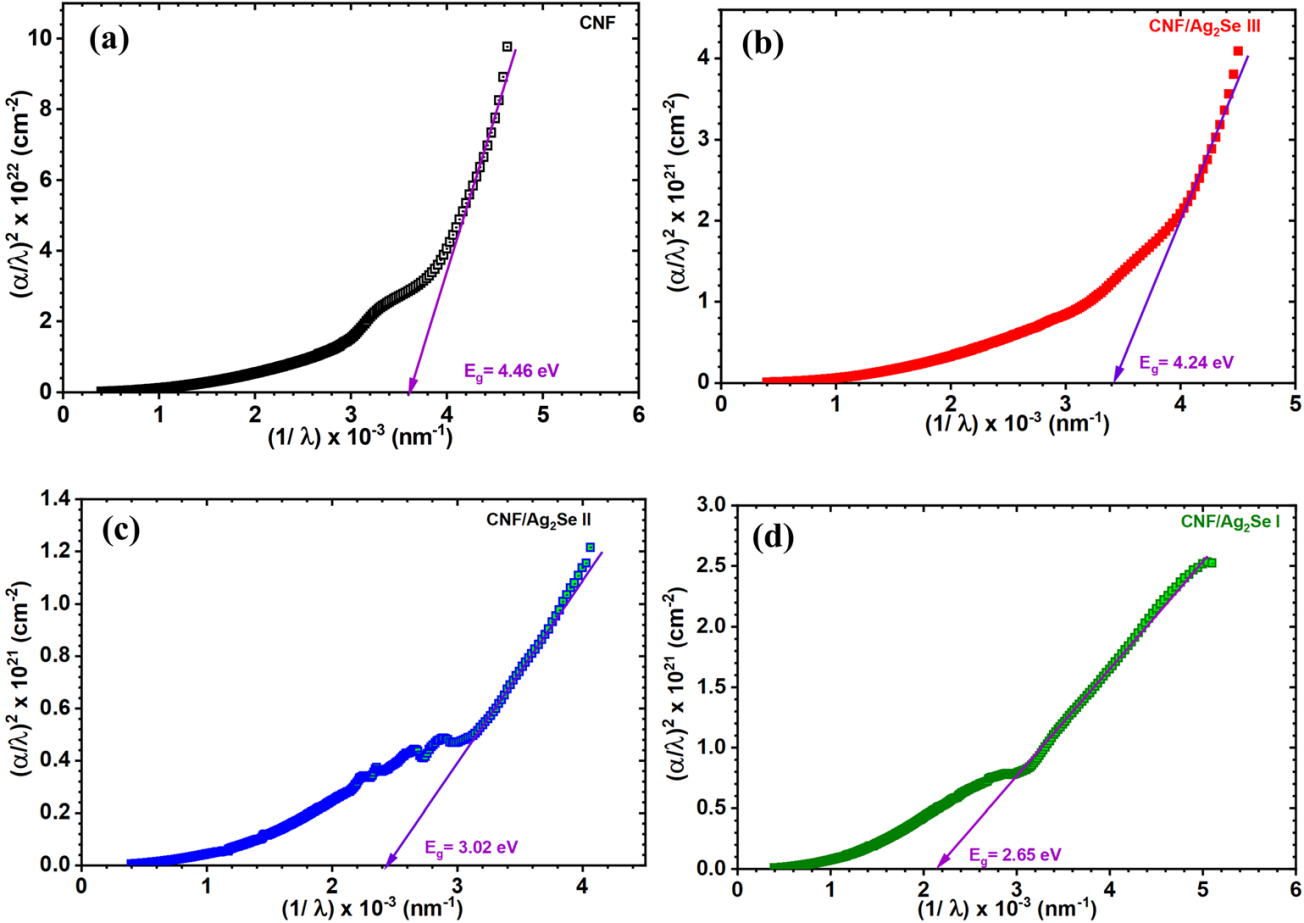
(a–d) Tauc plot ((*αhν*)^2^) *versus* photon energy (*hν*) for CNF, CNF/Ag_2_Se III, CNF/Ag_2_Se II, and CNF/Ag_2_Se I, respectively.

**Fig. 7:**
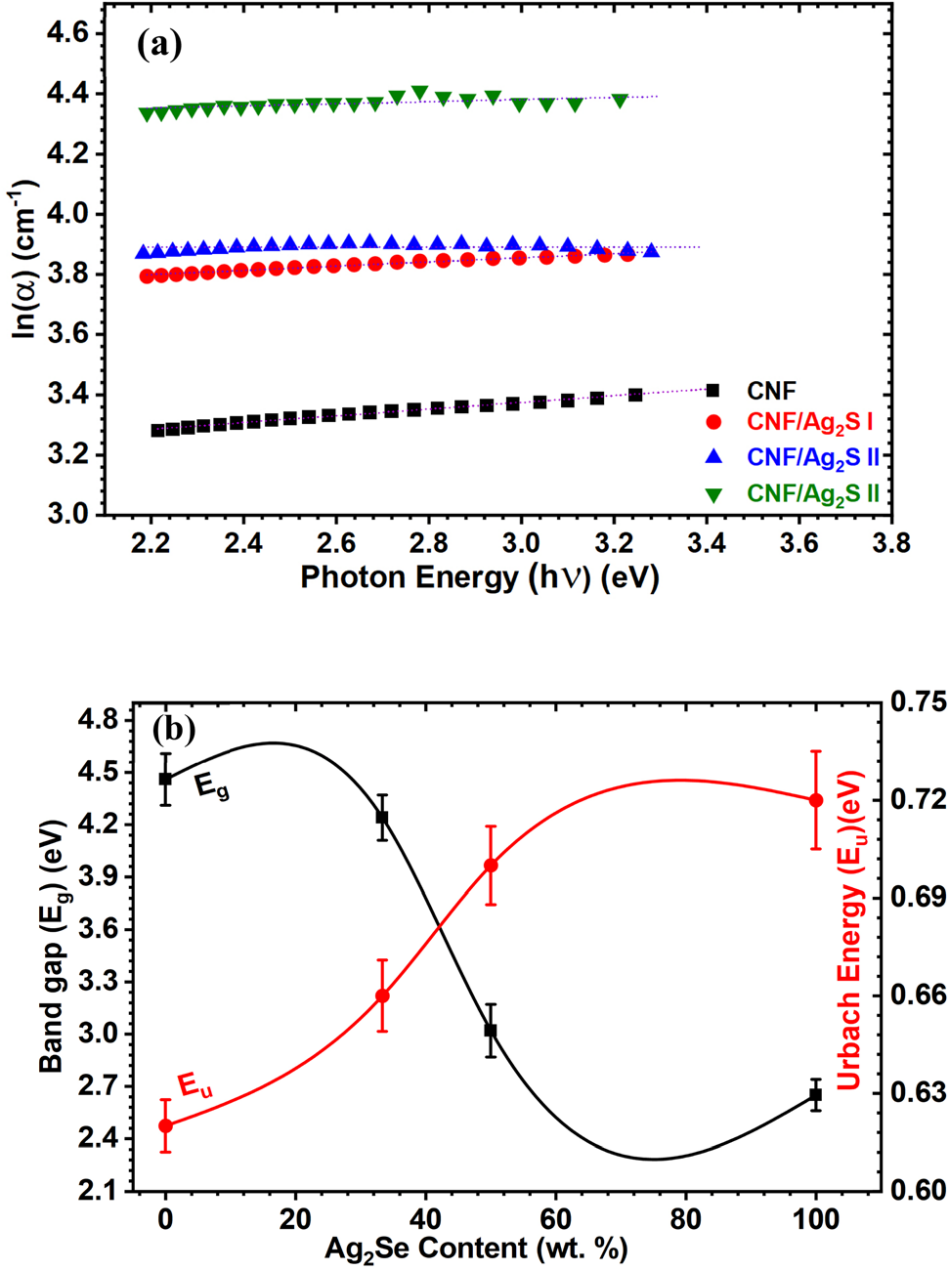
(a) Urbach's plot and (b) graphical representation of the variation in direct optical band gap and Urbach energy for CNF, CNF/Ag_2_Se III, CNF/Ag_2_Se II, and CNF/Ag_2_Se I.

The absorption spectra fitting (ASF) technique can be utilized by replacing the absorption coefficient (*α*) in eqn (6) and expressing the resulting relationship in terms of wavelength and absorbance. All the steps of this method have been stated in our previous reports.^33,34^ Therefore, the final relation between wavelength and optical band gap can be viewed as follows.6
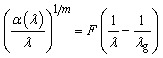
Here, *λ*_g_ denotes the wavelength that corresponds to the optical band gap. Fig. 6 a–d portrays the spectral representation of (*α*(*λ*)/*λ*)^1/*m*^ against 1/*λ* for pure CNF and the Ag_2_Se-modified CNF. The bandgap values were determined by graphically extrapolating the linear region to the wavelength axis, in accordance with the above equation. The optical band gap values for CNF, CNF/Ag_2_Se III, CNF/Ag_2_Se II, and CNF/Ag_2_Se I are 4.46, 4.42, 3.02, and 2.65 eV, respectively. The variation in the optical band gap with the Ag_2_Se content is portrayed in Fig. 7b. Accordingly, the optical band gap decreased upon increasing the content of silver selenide. The optical band gaps of CNF/Ag2Se II and CNF/Ag_2_Se I are within the visible region and at the optimal values, which indicate that they can be widely used in optoelectronic applications. The decrease in the band gap asserts that the Ag_2_Se material succeeds in making a strong intercalation with CNF thanks to situ preparation. The reduction of the optical band gap can be assigned to many factors, such as the introduction of localized states within the forbidden region and intervalence charge-transfer (IVCT) transitions. The formation of localized states forms a tail at the LUMO and HOMO, known as the Urbach energy, which is a good expression of the structural defects and degree of disorder within the band gap. The width of this tail can be assessed by Urbach's expressions, as stated below:

7 *α* = *α*_o_ exp(*hν*/*E*_u_)

The material-specific parameter *α*_o_ and the photon energy *hν* are used to determine the Urbach energy (*E*_u_) from the ln(*αhν*) against *hν* plot, as shown in Fig. 7a. Interestingly, the band tail was found to increase with decreasing band gap, as displayed in Fig. 7b.

### 3.5 Electrical properties of the T-CNF/Ag_2_Se films for energy-storage applications

#### 3.5.1 Dielectric spectroscopy

**Fig. 8:**
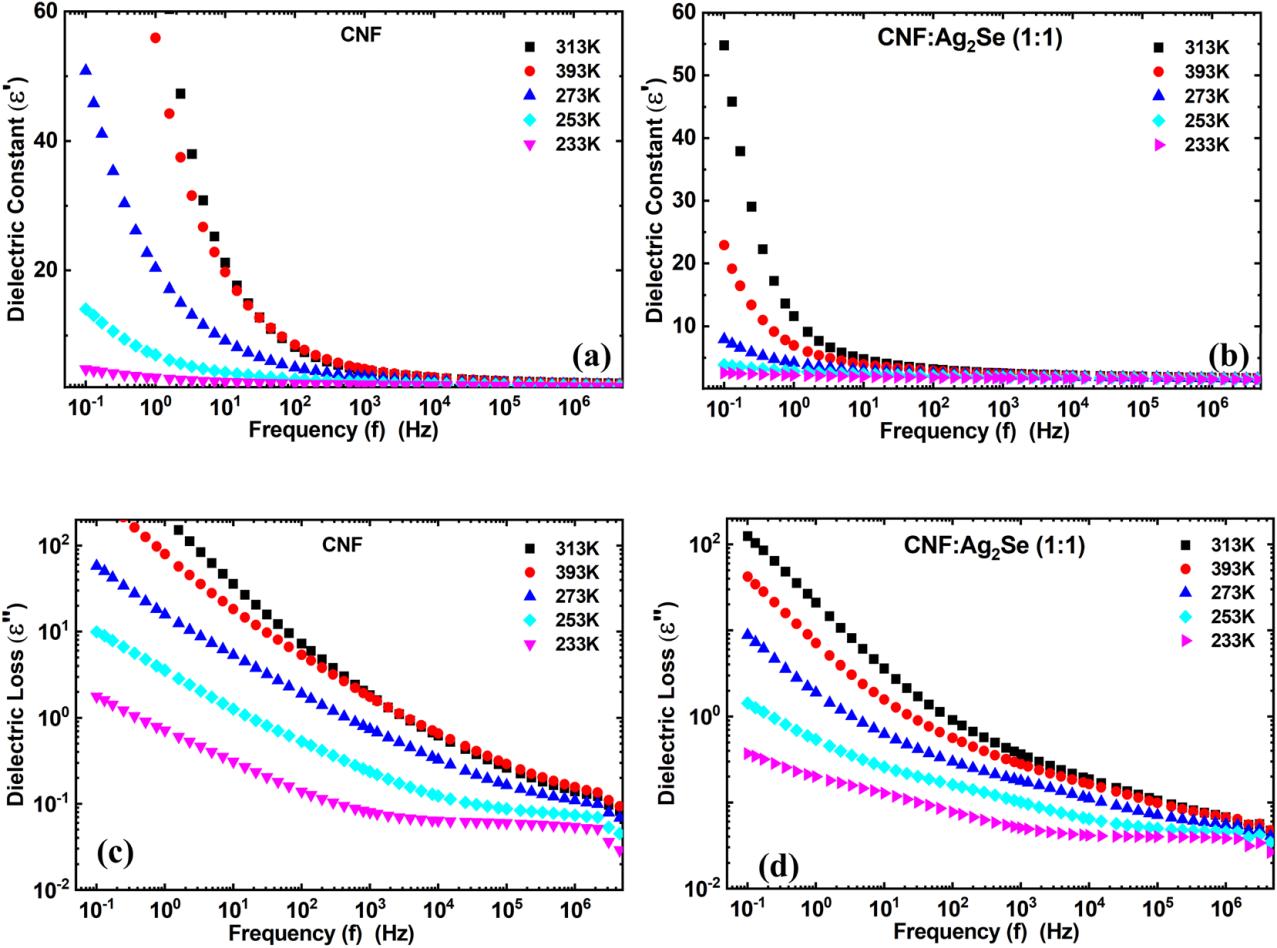
Dielectric constant of (a) CNF and the (b) CNF/Ag_2_Se I nanocomposites at different temperatures. Dielectric loss of (c) CNF and the (d) CNF/Ag_2_Se I nanocomposites at different temperatures.

**Fig. 9:**
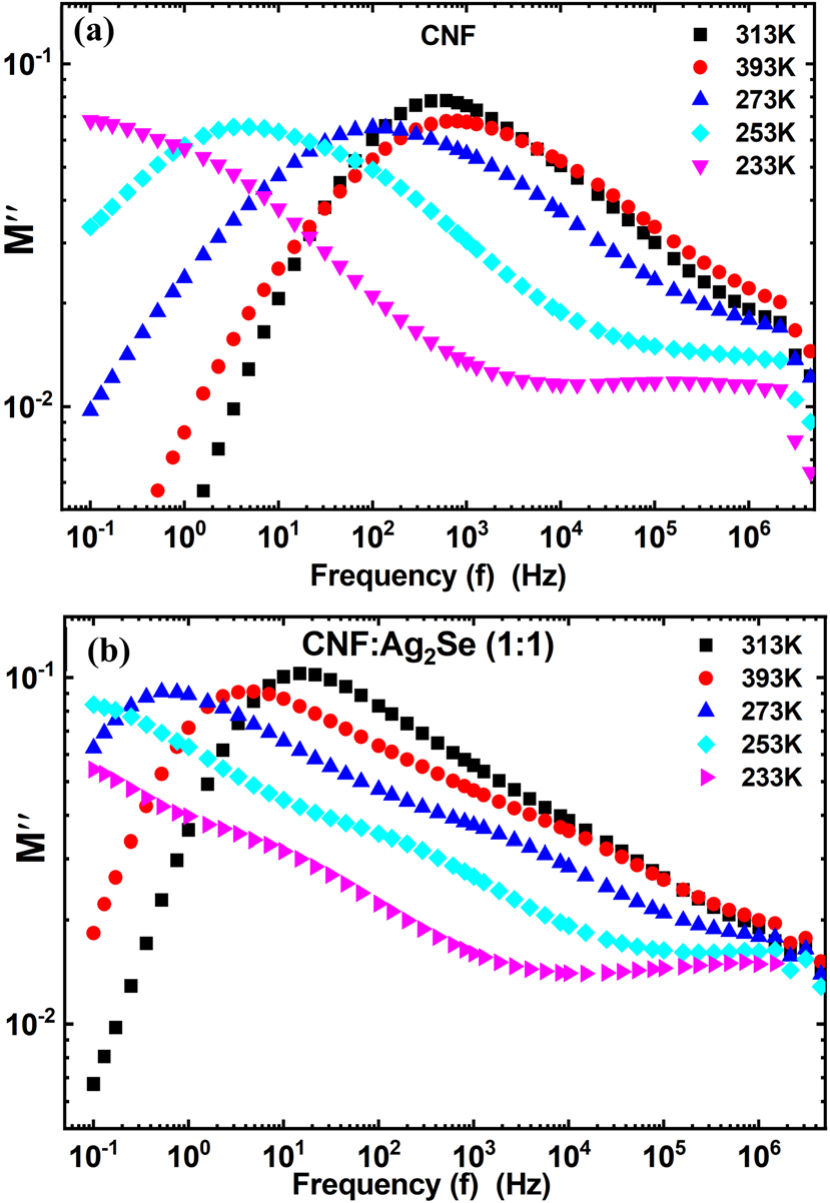
Variation in the imaginary part of the electric modulus (M″) with frequency for (a) CNF and the (b) CNF/Ag_2_Se I nanocomposites at different temperatures.

Fig. 8 and 9 illustrate the frequency- and temperature-dependent dielectric constant (*ε*′) and dielectric loss (*ε*″) of pure cellulose nanofiber (CNF) and CNF/Ag_2_Se (1 : 1) nanocomposite films. From Fig. 8a, it is clear that at low frequencies, the dielectric constant (*ε*′) of pure CNF is considerably higher, attaining values above 50 at 393 K. For all measured temperatures, *ε*′ decreases rapidly with increasing frequency. This trend is compatible with the well-known theory that dipoles, such as hydroxyl groups (–OH) in nanocellulose, can follow the applied electric field at low frequencies, thus producing strong polarization. Dipoles lag behind the oscillating field as frequency rises, hence the reduction in (*ε*′).^35^ When Ag_2_Se is included in a 1 : 1 ratio (Fig. 8b), the dielectric constant of the pure CNF across all frequencies and temperatures is much lower than those of the other materials. Still, (*ε*′) exhibits a frequency- and temperature-dependent behavior, though less pronounced. Ag_2_Se disturbs the continuous dielectric medium, thereby lowering the degree of polarization; also, the suppression of interfacial polarization by the restriction of charge build-up at the CNF/Ag_2_Se interface may occur.^36^ This reduced permittivity in CNF : Ag_2_Se can be attributed to the lower degree of polarization.^37,38^ The Maxwell–Wagner–Sillars interfacial polarization, originating from the free charges trapped at the heterogeneous interfaces among the insulating cellulose, conductive Ag_2_Se, and surrounding polymer matrix,^36^ is linked to the high (*ε*′) values at low frequencies for CNF, especially at higher temperatures. Under a slowly changing field, these charges can polarize, but at higher frequencies, they cannot relax rapidly, which causes a drop in (*ε*′). In particular, in the low-frequency range, (*ε*′) likewise increases for both materials as temperature rises. Enhanced thermal activation explains this by increasing both the mobility of dipoles and side groups (*e.g.*, from cellulose) and the orientation polarization.^39,40^ Characteristic of polar polymer matrices, this thermally induced polarization results from the molecular chain movement becoming more evident at higher temperatures.^41^ Interestingly, dielectric permittivity is known to be enhanced by aligned structures or conductive fillers, such as Ag-coated cellulose nanofibers, which mimic the mixture structure of CNF : Ag_2_Se, thus reducing dielectric loss. The alignment of conductive phases in an insulating matrix enhances the dielectric response *via* the simulation of an ideal two-phase parallel structure, claims Dang *et al.*^36^ The homogeneous CNF : Ag_2_Se mixture probably benefits from local conductivity paths that support this behavior, even though the conductive phases are not explicitly aligned in this system. The dielectric loss properties (*ε*″) for CNF (a) and CNF : Ag_2_Se (b) are shown in Fig. 8. With peak losses noted at low frequencies and high temperatures, (*ε*″) shows a strong decrease with increasing frequency in both materials. A high dielectric loss at low frequencies for CNF is the consequence of ion polarization, space-charge conduction, and hopping of charge carriers, events more active under low-frequency electric fields.^42^ The inability of ions and induced dipoles to react to the fast-changing field causes the sharp drop in (*ε*″) at higher frequencies, suppressing both conduction and vibrational polarization mechanisms. The CNF : Ag_2_Se composite shows a regularly smaller dielectric loss over all frequencies and temperatures than pure CNF. This behavior implies that Ag_2_Se lowers conductive loss paths and charge accumulation, thereby improving energy efficiency in dielectric applications. This is similar to the behavior recorded for aligned SFC (silver-filled cellulose) composites, in which the structured filler orientation attained a balance of high permittivity and low loss.^43^ Applications, including in electronics and energy storage, benefit especially from this reduction of dielectric loss. As a disadvantage, systems where the conductive paths exceed the percolation threshold^44^ exhibit a high (*ε*″), generating too much thermal energy under an electric field. Nevertheless, dielectric loss in the CNF : Ag_2_Se system is low even at high temperatures, implying that the composite is either operating below the percolation threshold or has a structure that prevents the formation of a conductive network. Furthermore, the decrease in (*ε*″) with frequency and its thermal activation at low frequencies indicate the presence but well-managed hopping mechanism and space charge effects in the composite.^42^

Fig. 9 shows, over a temperature range of 233–393 K, the variation of the imaginary part of the electric modulus (M″) with frequency for CNF (Fig. 9a) and CNF : Ag_2_Se (1 : 1) nanocomposites (Fig. 9b). A critical understanding of the relaxation behavior, space charge dynamics, and charge-carrier mobility in these nanocomposite systems comes from the electric modulus spectra. (M″) values are rather small for both CNF and CNF : Ag_2_Se at low frequencies.^45^ (M″) shows a characteristic relaxation peak that moves towards higher frequencies with rising temperature. This change represents a thermally activated relaxation mechanism whereby faster dipolar and charge-carrier reorientation is facilitated by increased thermal energy. Such temperature-induced changes in the (M″) peaks are commonly linked with the non-Debye type relaxation and hopping conduction mechanisms.^45–47^ The observed asymmetric peak forms support this by implying that structural or interfacial heterogeneity drives a distribution of relaxation times rather than a single Debye relaxation time. The electric modulus's relaxation peaks match a change from long-range charge carrier mobility at low frequencies to localized or limited movement at higher frequencies. While the right-hand side reflects the carriers' limited inside potential wells, the left side of the (M″) peak is ascribed to the carriers moving over longer distances.^45^ This behavior, particularly under higher-frequency fields, corresponds with the idea of conduction transforming from extended (long-range hopping) to localized (caged) motion.^48^ One can clearly distinguish CNF from CNF : Ag_2_Se. The (M″) peaks in the spectra of the CNF : Ag_2_Se nanocomposites are more broadly and methodically shifted toward higher frequencies than those in the spectra of the neat CNF. This change shows improved charge carrier dynamics enabled by the Ag_2_Se nanoparticles, which create extra conductive channels and support a more effective network for charge migration. This is consistent with previous results, where the addition of ZnO nanoparticles produced comparable (M″) peak shifts and enhanced charge mobility by means of a 3D conductive network generated inside the polymer matrix.^49,50^ (M″) tends to level off at high frequencies, suggesting that the carriers cannot follow the fast oscillating field. At this point, the almost constant (M″) values point to the system moving to localized relaxation, in which case only short-range reorientations or vibrational motions are feasible.^51^

The behavior of the CNF and CNF/Ag_2_Se nanocomposites closely matches what is observed in ZnO–PMMA systems, in which (M″) increases with frequency due to long-range hopping, peaks at a relaxation frequency, and then declines as carriers become confined.^48^ Likewise, in ZnO- or BT-loaded nanodielectrics, increasing the filler content improves charge transport by generating interfacial areas supporting Maxwell–Wagner polarization and allowing simpler ion migration, mirrored by the movement of (M″) peaks toward higher frequencies.^48,52^

The present CNF : Ag_2_Se system also reflects these trends since Ag_2_Se functions similarly to ZnO or BT, so it improves charge carrier dynamics and supports interfacial relaxation mechanisms. Moreover, the thermally activated character of the relaxation shown in Fig. 9 is compatible with the results of other polymer-based systems, such as PVA or PMMA composites, where the (M″) peaks shift with temperature due to the increasing mobility of trapped or interfacial charges.^53^

**Fig. 10:**
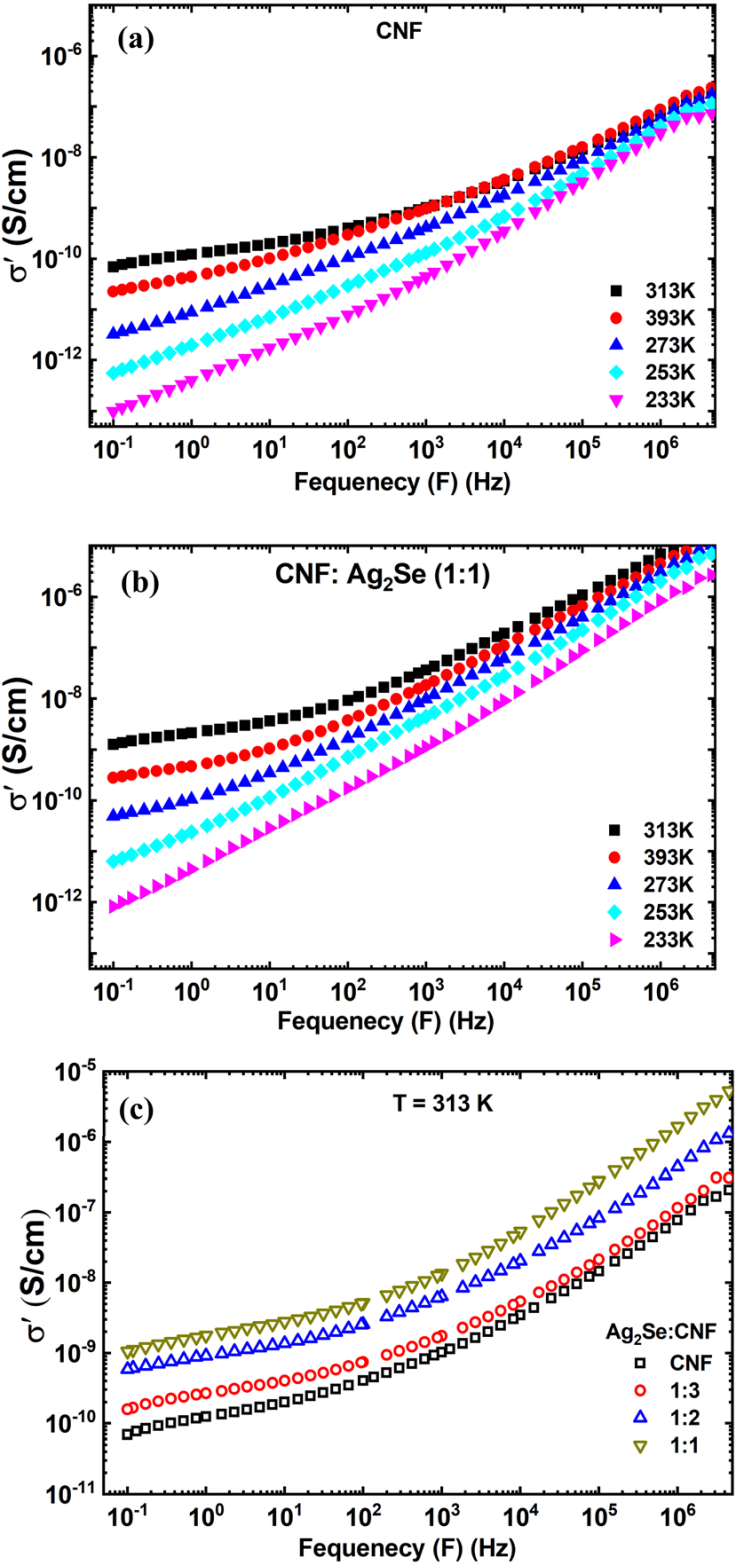
. Frequency dependence of the real part of the electrical conductivity (*σ*′) of CNF/Ag_2_Se I at (a and b) different temperatures and (c) 313 K. (In figure C, the content of CNF or Ag_2_Se is increased.

**Fig. 11:**
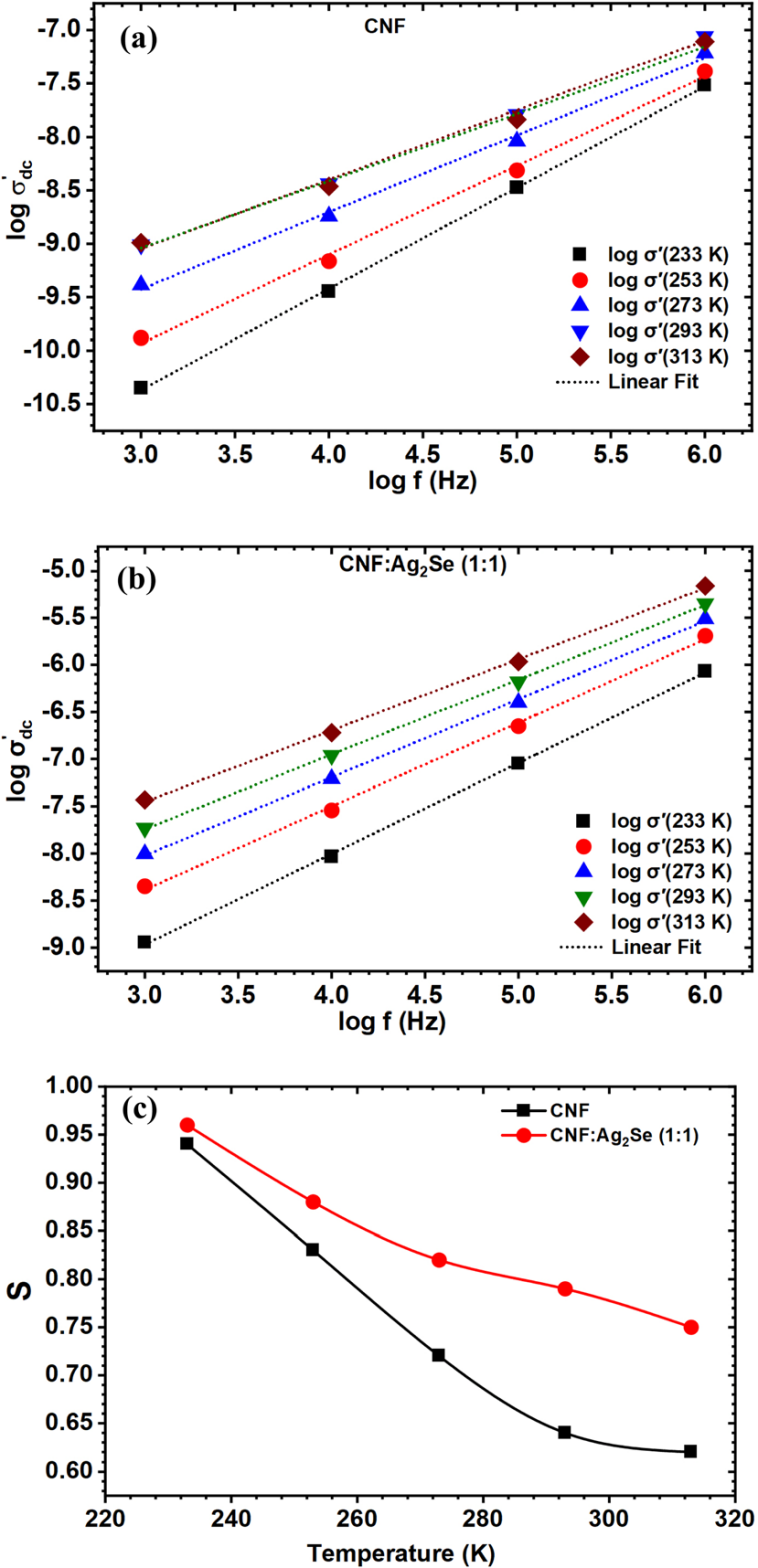
Frequency dependence of the DC conductivity for the (a) CNF and (b) CNF : Ag_2_Se (1 : 1) films, plotted as log *σ*′(*f*) *versus* log *f* at different temperatures. (c) Temperature dependence of the frequency exponent (*S*) for the CNF and CNF/Ag_2_Se (1 : 1) films at different temperatures.

Fig. 10a and b show the real part of the electrical conductivity (*σ*′) for CNF and CNF : Ag_2_Se nanocomposites as a function of frequency at various temperatures. Fig. 11c shows the CNF : Ag_2_Se ratio at 313 K. In all cases, *σ*′ rises with both frequency and temperature in line with the behavior of disordered systems controlled by the power law relationship.^34,54,55^

8 *σ*_T_ = *σ*_AC_ + *σ*_DC_ = *σ*_DC_ + *Aω*^*S*^

Here, *A* is the pre-exponential factor, *S* is the power law exponent (*S* > 1), *σ*_AC_ denotes the AC-electrical conductivity, *σ*_DC_ represents the DC limit of conductivity at *ω* = 0, A signifies constants indicative of polarizability, *ω* refers to angular frequency, and *S*(*T*) is the frequency exponent. The values of *S* fluctuate between 0 and 1, indicating the extent of interaction between mobile ions and their environment.^56^*S* is a variable influenced by the temperature and doping concentration, providing insights into the appropriate mechanism underlying AC-electric conductivity. This relationship, sometimes referred to as the “fractional power law,” shows that whereas at higher frequencies, AC conduction becomes prominent due to hopping or polarization effects, at low frequencies, the response is dominated by DC conductivity. This trend is widely observed in heterogeneous materials, including polymer/filler composites, ion-conducting glasses, and amorphous semiconductors.^57–59^ As the frequency rises, the frequency-dependent behavior noted marks a change from DC conduction to dispersive AC behavior. Conductivity stays almost constant at low frequencies, implying a DC plateau; at high frequencies, it increases dramatically, reflecting more hopping conduction of charge carriers. Slower charge carriers cannot follow the rapidly alternating field, which explains the frequency-induced dispersion results.^60^ The transition is connected to the distribution of relaxation times, typical of heterogeneous systems, where conductivity becomes progressively influenced by localized charge hopping or interface polarization processes.^61^ Conductivity is considerably affected by temperature as well. Higher (*σ*′) values for both CNF and CNF : Ag_2_Se composites are observed with rising temperature, as Fig. 10a and b show. This action follows Arrhenius-type thermally activated transport, in which temperature increases the free volume inside the polymer, enabling more segmental mobility and hence charge carrier migration.^62^ In particular, in systems including conductive fillers like Ag_2_Se, the higher ion mobility and the development of more conductive paths can be responsible for the increasing conductivity with temperature. The NFC/PVP/AgNP systems validate these results since they display higher conductivity with an increase in both the AgNP content and temperature.^63^

Fig. 10c underlines even more the Ag_2_Se filler's contribution to improving conductivity. The conductivity at 313 K considerably increases as the CNF : Ag_2_Se ratio rises from 1 : 1 to 1 : 3. The Ag_2_Se nanoparticles, acting as hopping sites or encouraging local charge accumulation at filler–matrix interfaces, help to create additional conductive paths and stronger interfacial polarization, which accounts for this behavior. Such interfacial effects resemble those seen in CNF-BT systems, in which better interfacial adhesion and charge movement along the nanoparticle perimeters enhance conductivity. Generally, the frequency- and temperature-dependent conductivity behavior of CNF and CNF:Ag₂Se nanocomposites indicates that the conductivity is enhanced with increasing temperature and filler content and hopping of charge carriers is the main conduction mechanism. Without producing continuous conductive networks, the addition of Ag_2_Se greatly increases (*σ*′) by increasing the charge carrier density and interfacial polarization. These characteristics imply that in antistatic or electrostatic dissipative (ESD) applications, where conductivity in the range of 10^−12^ to 10^−5^ S cm^−1^ is needed, CNF : Ag_2_Se nanocomposites could be valuable for this kind of application.^64^

For a more quantitative analysis, the AC conductivity data were examined within the Jonscher's framework by plotting log *σ*′(*f*) *versus* log *f* at each temperature and fitting the high-frequency region with a power-law dependence. The experimental curves in Fig. 11a for CNF and Fig. 11b for CNF/Ag_2_Se (1 : 1) deviate slightly from a single straight line over the whole frequency window, indicating that, strictly speaking, a modified Jonscher expression with more than one dispersive contribution would describe the data more accurately. Nevertheless, in the high-frequency range where a clear linear behavior is observed, a reliable effective exponent S can be extracted from the slope of log *σ*′(*f*) *vs.* log *f*. The resulting *S* values lie between about 0.6 and 0.97 for both the CNF and CNF/Ag_2_Se (1 : 1) films, which is consistent with hopping-type conduction in polymer and nanocomposite systems.^65^

Fig. 11c displays the temperature dependence of the frequency exponent *S* for the neat CNF and CNF/Ag_2_Se (1 : 1). In both systems, *S* decreases monotonically with increasing temperature, starting from values close to unity at 233 K and reaching approximately 0.6–0.75 at 313 K. This pronounced negative slope of *S*(*T*) is characteristic of the correlated barrier hopping (CBH) model and rules out tunnelling-dominated mechanisms (NSPT/QMT) (non-overlapping small polaron tunneling/quantum mechanical tunneling), for which *S* is typically nearly temperature-independent or even increases with temperature.^66^ Within the CBH model, charge carriers hop between localized states over potential barriers whose effective height decreases as the thermal energy increases,^67,68^ leading to an enhancement of the hopping probability and a concomitant reduction of exponent S. Similar decreasing S(T) trends and their interpretation in terms of CBH-type conduction have been reported for aromatic polyimide/CeO_2_ nanocomposites, magnetite nanoparticles Fe_3_O_4_, and PPy/(PhSe)_2_ nanocomposites.^66,69^ The slightly higher S values observed for CNF/Ag_2_Se compared to those for the neat CNF at a given temperature indicate that the incorporation of the Ag_2_Se nanoparticles modifies the distribution and height of potential barriers while preserving the same CBH-driven hopping mechanism in the cellulose-based matrix.^69^

### 3.6 Antimicrobial evaluation of the CNF/Ag_2_Se nanocomposites

**Fig. 12:**
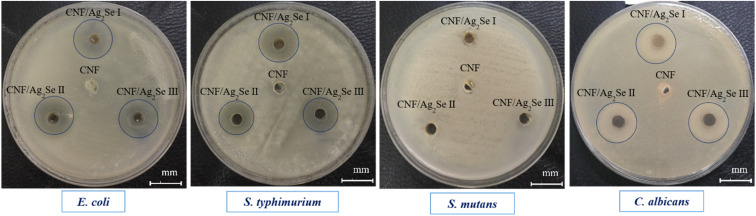
Antimicrobial activity expressed as the halo-zones of the CNF, CNF/Ag_2_Se III, CNF/Ag_2_Se II, and CNF/Ag_2_Se I nanocomposites against four pathogenic microbes.

The agar well diffusion method was used for evaluating the antimicrobial performance of the CNF, CNF/Ag_2_Se I, CNF/Ag_2_Se II, and CNF/Ag_2_Se III nanocomposites against four pathogenic microbes. After the incubation period of the pathogenic microbes, the diameter of the inhibition zone was recorded in order to investigate the release potency of the active agent of Ag_2_Se and hence its antimicrobial activity. The antimicrobial activity of the nanocomposite samples is presented in Table 2 and Fig. 12, and the results reveal that CNF/Ag_2_Se at different concentrations exhibited antimicrobial activity against *E. coli*, *S. typhimurium,* and *C. albicans* compared to CNF as a negative control. The results exhibit no significant activity against Gram-negative bacteria and *C. albicans*. Similarly, at higher concentrations, Ag_2_Se exhibits no significant antimicrobial activity, which is in line with a previous study.^70^*S. typhimurium* exhibited notable resistance against all the nanocomposite samples, as no inhibition zone was observed. From the obtained data, the effect of the CNF against all pathogenic microbes used was negligible, which is in agreement with other reported studies.^71–73^ This indicates that the antimicrobial response of each of the nanocomposite samples is due to the Ag_2_Se. The antimicrobial action of Ag_2_Se possibly deteriorates the cell membrane and disrupts the indirect oxygen reduction reaction (ORR), forming hydrogen peroxide (H_2_O_2_) oxidative species^74^ that interact with microbial DNA and proteins to prevent microbial replication, leading to cell death.^21^

**Table 2:** Antimicrobial activity of the CNF, CNF/Ag_2_Se I, CNF/Ag_2_Se II, and CNF/Ag_2_Se III nanocomposites against the four pathogenic microbes considered in this study

Pathogenic microbes	Diameters of inhibition zone (mm)			
	CNF	CNF/Ag_2_Se I	CNF/Ag_2_Se II	CNF/Ag_2_Se III
*E. coli*	0.0 (±0.0)	20 (±1.23)	19 (±1.23)	18 (±1.09)
*S. typhimurium*	0.0 (±0.0)	19 (±1.15)	18 (±1.33)	17 (±1.46)
*S. mutans*	0.0 (±0.0)	0.0 (±0.0)	0.0 (±0.0)	0.0 (±0.0)
*C. albicans*	0.0 (±0.0)	19 (±0.06)	18 (±1.17)	18 (±0.07)

## 4 Conclusions, outlook, and future aspects

TEMPO-cellulose/silver selenide (T-CNF/Ag_2_Se) nanocomposites were successfully developed by the *in situ* preparation of Ag_2_Se particles on TEMPO-oxidized cellulose nanofibers. Varying the silver and selenium concentrations produced uniformly dispersed Ag_2_Se particles with a narrow size distribution in the TEMPO-oxidized cellulose nanofibers. XRD verified the successful inclusion of Ag_2_Se into the CNF polymer *via* the appearance of new peaks related to Ag_2_Se and proved that the degree of crystallinity, as well as grain size, was ameliorated, indicating improved structural and functional properties suitable for advanced optoelectronic and energy-related applications. FTIR confirms the successful modification of CNF with Ag_2_Se. The presence of Ag_2_Se affects both the fingerprint and hydroxyl regions, demonstrating interaction between the filler and polymer, which can influence mechanical, optical, and electronic properties. Further affirmation of the strong intercalation between CNF and Ag_2_Se was asserted by the great variation in the optical properties, including transmittance, reflectance, optical band gap, and Urbach energy (generated disorders). HRTEM images establish the presence of an enhanced hierarchical structure *via* the existence of twisted or curved configurations, which is beneficial for the electrical properties. The optical band gap decreases from the UV region for CNF (4.46 eV) to the visible light region (3.03 and 2.65 eV, for CNF/Ag_2_Se II and CNF/Ag_2_SeI, respectively), establishing the validity of the polymeric films for photocatalyst and optoelectronic applications. The electrical properties proved that the incorporation of Ag_2_Se nanoparticles into cellulose nanofibers significantly enhances charge carrier dynamics, increases dielectric loss, and improves AC conductivity through interfacial polarization and hopping mechanisms. The CNF : Ag_2_Se nanocomposites exhibit thermally activated, frequency-dependent dielectric behavior, demonstrating superior energy efficiency and stability compared to the pure CNF. These results highlight the potential of CNF : Ag_2_Se composites for advanced electronic and electrostatic dissipative applications, where controlled conductivity and low dielectric loss are critical. The data obtained suggest that the nanocomposite materials investigated have a better inhibitory response against Gram-negative bacteria and *C. albicans*, which confirm that CNF/Ag_2_Se may be useful in the biomedical sector.

## Author contributions

Ahlam I. Al-Sulami: conceptualization, methodology, data curation, formal analysis, writing – original draft, validation, investigation, writing – review & editing, visualization: data curation, formal analysis, validation, and investigation, writing – review & editing. Fatimah Mohammad H. Al Sulami: conceptualization, methodology, data curation, formal analysis, writing – original draft, validaion, investigation, writing – review & editing, visualization: data curation, formal analysis, validation, and investigation, writing – review & editing. Reema H. Aldahiri: conceptualization, methodology, data curation, formal analysis, writing – original draft, validation, investigation, writing – review & editing, visualization: data curation, formal analysis, validation, and investigation, writing – review & editing. A. Lahmar: conceptualization, methodology, data curation, formal analysis, writing – original draft, validation, investigation, writing – review & editing, visualization: data curation, formal analysis, validation and investigation, writing – review & editing. J. Zidani: conceptualization, methodology, data curation, formal analysis, writing – original draft, validation, investigation, writing – review & editing, visualization: data curation, formal analysis, validation and investigation, writing – review & editing, Talaat A. Hameed: conceptualization, methodology, data curation, formal analysis, writing – original draft, validation, investigation, writing – review & editing, visualization: data curation, formal analysis, validation and investigation, writing – review & editing.

## Conflicts of interest

The authors declare that they have no conflicts of interest. The present results and discussions presented in this submission are original. This research was not funded by any authority, entity, or individual other than the authors themselves. They bear all the costs of the work.

## Data Availability

The authors confirm that the data supporting the findings of this study are available within the article. Raw data that support the findings of this study are available from the corresponding authors upon reasonable request.
